# Applying FAIR4RS principles to develop an integrated modeling environment for the magnetic confinement fusion

**DOI:** 10.1038/s41597-023-02470-y

**Published:** 2023-09-07

**Authors:** Xiaojuan Liu, Zhi Yu, Nong Xiang

**Affiliations:** 1 University of Science and Technology of China, Hefei, 230026 China; 2 Institute of Plasma Physics, Hefei Institutes of Physical Science, Chinese Academy of Sciences, Hefei, 230031 China

**Keywords:** Magnetically confined plasmas, Nuclear fusion and fission

## Abstract

Over the decades, the integrated modeling (IM) environment for magnetically confined fusion has evolved from a single, isolated, proprietary numerical computing software to an open, flexible platform emphasizing sharing, communication, and workflow. This development direction is consistent with the FAIR4RS principles put forward by the scientific community in recent years. In this article, we describe how the FAIR4RS principles were put into practice during the development of the IM management tool FyDev for the Experimental Advanced Superconducting Tokamak (EAST). FyDev integrates the process of building, deploying, and invoking research software, automating the entire process. FyDev can also assign a unique ID for each software, convert the software ID to a Python module, and encapsulate a package management tool to enhance the software building process, ensuring consistency throughout the entire phase of the research software find, access, use, and invocation in a uniform contextual environment.

## Introduction

Like many modern scientific research projects, magnetic confinement fusion devices (i.e., tokamaks) are complex systems that involve multiple physical processes and scales of space and time. Their modeling and analysis require several research software packages with different functionalities to work together, so-called integrated modeling and analysis (IM). To better understand the experiments, we need more comprehensive and integrated research software packages that cover a wider range of physical processes. A typical tokamak experimental analysis process invokes multiple physics research software^1^. For decades, the IM environment for magnetic confinement fusion has evolved from the single, isolated, proprietary numerical computation software to the open, flexible platform emphasizing sharing, communication, and workflow^1–7^.

This development direction is in keeping with the contemporary scientific research community’s demand for widespread sharing and collaboration of scientific data. The 2016 article “FAIR Guiding Principles for scientific data management and Stewardship”^8^ presents guiding principles for managing scientific digital assets Findability, Accessibility, Interoperability, and Reusability (FAIR). This article states, “Good data management is not a goal but rather the key conduit leading to knowledge discovery and innovation, and subsequent data and knowledge integration and reuse by the community after the data publication process.” The European Fusion Community has launched the Fair4Fusion project^9^ to enhance fairness and openness in research and to increase accessibility and sharing of fusion data. However, the transparency of data origins and reproducibility of data results are often overlooked, especially with the large amount of simulation data generated by IM. These are determined by the computing environment that produces and analyzes data isolated from the researchers outside the IM system. As the article^10^ explains, the computing environment used to run the entire data analysis is often skipped or incompletely described in scientific analysis reports, seriously affecting transparency and reproducibility.

The IM software environment is particularly suitable for this scenario. The complex research software is the component that makes up the IM environment. It comprises complex computing environments consisting of research software and related dependent software libraries. It is particularly important in IM and cannot be ignored. We need to obtain new physics conclusions from data through modeling and analysis software. New theories and models must be applied to research through software. For most application scenarios, the software is often built using other software. The management of research software is just as critical as data management and should receive more attention. Recently, the FAIR Principles for Research Software (FAIR4RS)^11–14^ have been proposed to address the challenges of research software management. The FAIR4RS principles require scientific software to be findable, accessible, interoperable, and reusable. Then, we can use the FAIR4RS principles to guide the development of software management tools suitable for IM. Following FAIR4RS principles, we can enhance the transparency of software in IM, especially the transparency of the build process, and provide more complete and rich metadata information, which can help researchers to understand the software and its dependencies better.

This article describes how the FAIR4RS principles were implemented during the IM management tool, FyDev, development for the Experimental Advanced Superconducting Tokamak (EAST)^15^. FyDev integrates the process of building, deploying, and invoking research software, automating the entire process. The reproducibility of the research software and the data from the calculations is thus guaranteed. FyDev can also assign a unique ID for each software, convert the software ID to a Python module, and encapsulate a package management tool to enhance the software building process, ensuring consistency throughout the entire phase of the research software find, access, use, and invocation in the uniform context environment.

## Results

The IM process for magnetic confinement fusion invokes and composes external software from different research directions in the community by building a unified architecture. These codes have inherent characteristics and were developed by different researchers in the fusion community over decades of research. IM for EAST uses Python as the glue language to manage the data exchange and workflow between the different research software.

**Fig. 1:**
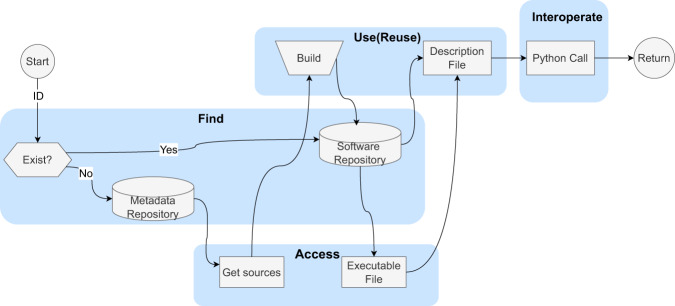
FyDev incorporates the four FAIR aspects into using research software in an IM process. The software ID is converted into a module in Python through the Find, Access, and Interoperate stages if the software is installed. Otherwise, the “Build” module is invoked to install and deploy the software in the Use (Reuse) stage. FyDev follows the FAIR4RS principle in the Python context by using a unified Application Programming Interface (API), standardized version management, and modular packaging to find, access, build, and invocate software.

In this work, we explore how to adapt the FAIR4RS principles to the management and use of research software in an IM for EAST from the perspective of the software user. High-Performance Computing (HPC) is a typical computing environment for IM and requires diverse software to support different physical research. We designed a prototype application invoked FyDev, which unifies different software into Python modules. FyDev automatically manages and documents the software build process, providing a uniform provenance for invokes. This allows the software in IM to be traceable from find, build, to execute. The process is divided into four stages, as shown in Fig. 1, which describes how FyDev incorporates the four FAIR aspects into using research software in an IM.

Firstly, the ID helps to find. Software repositories store binaries and description files for deployed software. Metadata repositories store metadata description information about software. ID supports retrieval for both repositories.

Secondly, the software ID can be converted into a Python package through the Find, Access, and Interoperate stages if the software is installed. Otherwise, the “Build” module directly builds the software in the runtime context, supplementing the missing build information in the traditional software process and avoiding inconsistent build environments independent of the runtime environment.

Finally, The software repository contains an executable and a local description file, which is released along with the executable. The local description file adds adaptive information about the generated binary file in the current runtime environment to the metadata template file. This creates a complete provenance record of the software, including its build, execution, and interaction details. The software runs as a Python module during the interoperability phase.

## Discussion

The FAIR4RS Working Group has proposed the Findable, Accessible, Interoperable, and Reusable (FAIR) principles for research software. The principles provide a foundation for optimizing the reuse of research software. FyDev serves as a prototype for FAIR4RS in Software Management to make the application of IM for EAST as FAIR-compliant as possible. Implementing the FAIR4RS principles in FyDev is listed in Table 1. The last column lists some information we think should be added to the FAIR4RS. FyDev focuses on the software usage perspective and does not consider the constraints on the software development process in the FAIR4RS principles, such as R1.1. ID, metadata, and the “Build” module are core components in FyDev. We will discuss them below in the context of the four aspects of FAIR.

**Table 1:** The table describes how FyDev implements the FAIR principles for research software in IM for EAST.

contents	Apply in FyDev	Enhanced
Findable	**F1:** The research software in IM is identified in FyDev with “name-version-toolchain-version-suffix”. The ID is humanly readable and machine-interpretable. The ID uniquely identifies the binary executable of software. **F2/F3:** Metadata file to record information about the research software including basic information, dependent components, build method, run method, IO parameters, etc. **F4:** Remote metadata repository for FyDev is based on GIT repository management. It serves as the source for heterogeneous platforms.	(1) The ID should contain the dependent component, identified by toolchain-version-suffix. (2)Adding build information to the metadata.
Accessible	**A1:** In FyDev, a unified API that uses ID as input is developed to access consistently binary executables in the software repository or access metadata to find the source code. **A1.2:** Software (binary files) access rights are determined by the User Identify (UID)/Group Identify (GID) administration in Linux system. **A2:** Metadata can ensure the long-term accessibility of the executable file, as long as the source code is not lost.	Consistent access and management of binary executables should be considered.
Interoperable	**I:** (1)The metadata and “Build” module address the interaction of the software with other software and the runtime environment. (2)The descriptions provide the standard templates for IO. (3)SpDB^21^ provides a unified API based on the standard data model.	The run command and run parameters should be recorded in the metadata.
Reusable	**R1:** The local description file details the provenance information. **R2:** The “Build” module improves the use of the software (reusable). **R3:** Software is converted to the standard wrapper module in Python.	The build process should be added to the runtime.

It describes how FyDev makes software findable, accessible, interoperable, and reusable which uses metadata files to record information about the research software, including basic information, dependent components, build method, run method, IO parameters, and more. FyDev has a unified API that uses ID as input to consistently access binary executables in the software repository or access metadata to find the source code. The local description file details the provenance information, and the “Build” module adds the build process to the runtime, which improves the use of other software (reusable). Finally, the software is converted to a standard wrapper module in Python, and the description file facilitates the software’s interaction. In the “Enhanced” column are given what we think should be highlighted in FAIR4RS. FyDev focuses on the use of the software. Therefore, some elements are not included, such as R1.1 (in FRAI4RS)on licensing and R3 (in FAIR4RS) on community standards in software development.

### Identifiers in FyDev

Findability is a fundamental principle that requires finding a resource before any other consideration. For research software, findability means ensuring the software can be unambiguously identified using common search strategies^11^. Identifiers (IDs) are a mechanism that allows for unambiguous identification of the referenced resources, thanks to their global uniqueness. The source code ID includes the source code name and version or a more general DOI^11^, which can be an ID for a generic software configuration. However, this is not enough. IDs should also be assigned to other related components and specific deployments to facilitate data provenance and reproducible research processes^11^. As explained in the article^10^, the source code needs to be built and deployed into an executable file, and the build and deployment process affects the behavior of the final executable file. The relationship between components should be reflected in the associated metadata. This allows for an identifier (ID) at a higher conceptual level for the software to be linked to its subcomponents^12^.

FyDev tries to improve the situation while being pragmatic about its actual application purpose. The ID, which is more common in FyDev, is *name-version-toolchain-version-suffix*. The *toolchain-version* is a collection of base packages of a specified version, including a compiler, parallel library, math library, data library, etc. The *suffix* indicates custom modifications and additional information. Regarding *name-version*, especially “*version*,” FyDev does not break the “*version*” tag or DOI for source code. FyDev only provides the mapping relationship between ID and software, adding relevant dependent component information to the mapping result. This information is necessary for software to become a binary file that users can use. This internal mapping mechanism of FyDev makes ID more human-readable.

The “global” for FAIR requirements are consistent across communities. FyDev continues the DOIs that already exist in the source code, but the uniqueness of the build process only makes sense in FyDev. Because FyDev can rebuild software according to ID in different environments and ensure consistency, while leaving FyDev cannot guarantee the consistency of the build process. That is to say, FyDev provides the mapping relationship between ID and software and does not undertake the function of ensuring “global” uniqueness.

Various mechanisms, such as hash codes, can guarantee uniqueness. It maps data of any length into fixed-length codes. It can be used as checksum information placed in metadata to identify source files uniquely. However, hash codes are not interpretable and lack readability. Moreover, hash codes can only identify the software itself but cannot reflect software dependencies and characteristics. Therefore, FyDev uses ID as an identifier for the delivery chain rather than the software itself. ID provides useful information about software name, version and toolchain, which can help users quickly identify and compare different software products. This is a trade-off between different needs, focusing on readability or uniqueness.

As a prospect, future versions will integrate a metadata hash to make FyDev more powerful. At the same time, we hope the community can reach a consensus and establish a centralized registration mechanism to maintain true global uniqueness further. The size of the developer community that recognizes this registration mechanism determines the effective scope of global uniqueness for ID. When this global mechanism is established, FyDev can easily establish the mapping relationship between the “internal” ID in FyDev and the “global” ID.

### Metadata and “Build” module in FyDev

FyDev’s purpose is to use the ID to locate the research software in IM and transform them into modular Python packages that can be invoked. The conversion process is more likely to comply with the FAIR principles with the help of metadata and the “Build” module.

FyDev’s metadata contains two aspects: (1) metadata repository. (2) description file in the software repository. A template, shown in Table 2, to display the metadata file’s main content for IM software. The local description file dynamically adds to the metadata template file the adaptive information of the generated binary file in the current runtime environment, forming a complete provenance record of the software.

**Table 2:** The metadata file of research software is presented as a Key-Value format in YAML.Some information in the file is static, and some are dynamically updated according to the computing environment, such as prescript and *install_dir*. A well-documented local description file is an upgraded version of the metadata that will eventually be stored on the repository along with the executable binary.

Most existing IM system separates the building and invokes research software. Users only care about the interface information of the software’s metadata record. The package management tool under the runtime environment only cares about the metadata record of the package-building process. The separation causes incomplete metadata records. In practice, users use binary packages already deployed without a clear understanding of the installation process. The same software requires adaptation to different runtime environments. In FyDev, metadata describes complete information of corresponding research software under the current usage environment, including software source code acquisition, component dependencies, compilation, installation, usage, and IO information records. Unlike a single package management tool, FyDev provides a way to extend traditional package management build tool metadata such as buildinfo^16^ for Debain, config-file for Guix^10^, easyconfig for EasyBuild^17^.

The “Build” module in Table 1 is added to FyDev to take care of the source of the binary distribution process. It adds the build process for research software in IM to the Python environment. It is based on metadata files. The “Build” module is usually independent of the software usage process but seriously affects its usability. Source code and version alone are insufficient to provide all the information needed for (re)deploying the computing environment, whether to meet current or future needs^10^. Software running on any computer results from one source code transformed into binary by another software (e.g., compiler). The process involves built-time dependencies and run-time dependencies. In FyDev, when the invoked software does not exist, it can be automatically compiled and installed in the current Python context environment, dynamically track and record the entire install process, and update it in the local description file for subsequent use.

The “Build” module parses the metadata file. It generates or directly obtains the installation configuration file for the underlying package management tool to install and deploy the research software. FyDev does not develop new package-building tools but rather chooses mature tools to fit IM application scenarios. Several package management tools have been developed to ease the burden of using complex scientific packages for scientists. A detailed comparison is given in Table 3. Combining the characteristics of scientific software in IM systems and scenarios, EasyBuild^17–19^, and Lmod (https://lmod.readthedocs.io/en/latest/010_user.html) for HPC were preferred as the default package-building tool encapsulated in the underlying FyDev. However, FyDev does not restrict the package-building tools. Flexible interfaces are reserved for code development. Later on, more package management tools can be incorporated according to the characteristics of the newly accessed application and user needs.

**Table 3:** The table compares a variety of established software management tools available regarding build method, support for multiple versions, dependency resolution, and flexibility of installation directories.

Type	container	system-based	Based on a distributed filesystem	Based on Linux kernel	Based on Lmod or environment module^25,26^
**Name**	Docker^27,28^, Apptainer (singularity)^29^	YUM or APT	CVMFS^30–32^	Guix^10^	EasyBuild^17^, Spack^34^
**Build method**	packaging an entire system into a static bundle, lower flexibility	binary packages	based on other tools, such as EasyBuild, Spack	binary packages	building from source
**Distributed, Multi-Version**		No	Yes	Yes	Yes
**Dependence**		Automatic	Support for manual customization, including compilation options and dependency modules	Automatic	Support for manual customization, including compilation options and dependency modules
**Install path**		Fixed	Fixed	Fixed	Flexible

EasyBuild is a software build and installation framework that allows you to manage (scientific) software on HPC systems efficiently. It is designed to easily install scientific software packages and their dependencies on HPC systems. Lmod is a Lua-based environment module system that easily handles the *modulepath* hierarchical problem. It simplifies managing software environments by allowing users to switch between different software package versions easily. The extensive Easyblock class in the EasyBuild tool, and the script-based easyconfig file specification, bring new inspiration and guidance to the management approach. In FyDev, the configuration file corresponding to the research software in each IM is standardized and customized according to the easyconfig file format. The filenames and versions are organized following the rules agreed upon in EasyBuild. This results in a proprietary configuration file repository for the software sets in IM. This repository is an extension to the file repository that comes with EasyBuild during the software build process. As a whole, it forms a collection of files that can automatically resolve dependencies. It is tracked in the folder (*FyEbfiles*) and is managed as a subset of the metadata repository (*FyModules*).

In FyDev, the metadata file and the “Build” module have complementary relationships. The interpretable metadata file specifies the software’s dependent runtime environment. On the other hand, the “Build” module is responsible for building the corresponding soft environment and managing the research software in the IM uniformly. The “Build” module’s dynamic construction process will automatically fill in more provenance information and ensure the software’s final executability and (re)usability.

### Findability

FyDev uses a local description file that matches the executable file individually to locate it. The local description file details the other components used to generate the executable and the interface information needed for subsequent invokes. In this way, finding the executable translates into finding the local description file. The local description file and the executable are stored in the software repository. Further, when the executable software does not exist, FyDev supports building it starting from metadata in the metadata repository.

Therefore, as shown in the Find phase in Fig. 1, the software IDs in the IM need to support the retrievability of the software repository and the metadata repository. First, the ID helps to look up the software repository. In FyDev, the query path for the software repository is agreed to be a directory structure in the form of *RootPath/software/fydev/physics/name*. The ID helps to locate the corresponding description files and executables directly. Secondly, the ID helps to locate the metadata repository. Similarly, the directory structure and file format of *RootPath/repository/{name}-{version}-{toolchain}{version-suffix}.yaml* is agreed upon. The metadata file contains information about the ID. When the software is not installed, the metadata file is parsed by subsequent stages to obtain the source code and generate executables. This design form can improve the target file’s retrievability by using the ID’s readability. In other words, it is a compromise between readability and uniqueness. This mechanism is effective in a single domain within FyDev’s usefulness. Mechanisms like DOI and hash are more accurate in a wider scope of use and access. FyDev can enable the conversion between the two methods of ID definition by defining “aliases” and so on.

### Accessibility

Different types of software have different access methods. In IM, research software is mainly developed and disseminated by researchers within the community. The source code is the most direct way to access it. The developer or the user ensures the distribution of the source code through various means, such as Git (https://git-scm.com/), Zip/7-Zip (https://www.7-zip.org/), etc. However, the metadata records the source code’s storage location and access method. The executables, the final form of using the research software in IM, are generated from the source code compilation. Therefore, the actual scenarios in IM involve the retrieval of both the software source code and the binaries, which are obtained from the metadata repository and the software repository, respectively, with the help of IDs as shown in Fig. 1.

1. Obtaining source code: If there is no pre-compiled software in the software repository, the software must be compiled from source code and deployed to the software library. We use *module.fetch(ID)* function to obtain the source code from the software’s description file. The source code location can be any network or local computer path. The subsequent process of obtaining source code will be explained in detail in the Reusability section.
2. Loading executable files: If the executable file which is accessed already exists in the software repository, it can be loaded through the *module.load(ID)*. This API will look for the local description file, parse the software’s preprocessing keyword inside the file and add the appropriate path to the current runtime environment.

In FyDev, we designed a simple API to encapsulate IDs and improve accessibility uniformly. The current version of the API does not involve authentication. The code developers determine the access rights to the source code. We adopt the Linux system’s UID/GID management method in the software environment to restrict permissions for executable files and their folders. User and group management in the HPC cluster implement authentication and authorization here.

In FyDev, metadata is a detailed description template of the general information of the software, which facilitates the software’s redeployment on different platforms. The description file is a provenance of the specialized information of the software that is converted into an executable file on the current platform. It is unique and facilitates the software’s reproduction and reuse on the current platform. From the perspective of access, the two levels of metadata can ensure the long-term accessibility of the executable file as long as the source code is not lost.

### Interoperability

Compared to data, the interoperability of research software has been recognized as one of the most challenging of the four FAIR principles. In particular, the concepts of interoperability and reusability are significantly more complex when discussing a dynamic behavior than tracking the behavior of the data objects in which these actions occur. Further, for software, interoperability directly affects the feasibility of reproducibility. Interoperability for the software itself is considered from three main viewpoints^20^:

1. The software is an independent operational object with intrinsic self-consistent structure, such as a suitable programming language, API, data format, etc., to produce a runnable software version.
2. The harmonious coexistence of the software and the runtime environment forms a set of stacks, including the software itself, the dependency environment, the execution environment, etc.
3. The interconnection and coupling of different software under the same the system through agreed protocols and standards.

The first item belongs to software development and is beyond the scope of this paper. The second item is categorized under software accessibility and usability, as described in the Accessibility section. The main challenge we face is the coupling of different research software due to their specific requirements.

The coupling relationship and method depend on the software’s functionality. We focus on achieving self-consistent integration between complex and diverse research software and their underlying environment within the same IM, given the predefined data I/O. To achieve this goal, we need to address two issues: (1) how to provide precise IO descriptions for each software, including file types, locations, names, and other information. (2) developing data conversion tools based on standardized semantic representations. We use description files and the SpDB^21^, a data integration tool with Data dictionary (DD) in Integrated Modelling and Analysis Suite (IMAS)^1^ as the standard data model to solve these two problems. The description file specifies each software’s content and data IO types, which helps with context interpretation. The description file and executable software correspond one-to-one and are maintained by FyDev. SpDB is a data integration tool that uses the input and output keywords provided in the description file to convert different types of source data into I/O operations under a standardized data model, facilitating software interaction. SpDB coexists with FyDev to support the IM environment, although it is not part of FyDev. With the help of the description file and SpDB, we can provide a unified API for different software and facilitate interaction between them.

### Usability (Reusability)

The software’s usability focuses on the ability of humans and clusters to execute, inspect, and understand the software so that it can be modified, extended, or integrated into other software. At its core, reusability aims for someone to re-use software reproducibly^22^. As demonstrated in arctile^23^, reproducibility is hard yet tremendously necessary. However, some simple measures can tremendously increase reusability and, at the same time, strengthen reproducibility and re-runnability over the long term. Documentation is one of the most potent tools for reusability. A comment describing what each function does, however evident, can avoid hours of head-scratching. So, comments and documentation can make a significant difference. From a developer’s perspective, sufficient documentation and comments during software development benefit users because it can be installed, run, and understood in a few hours. The software cannot exist independently and needs to interact with other software. From a user’s perspective, the maintainability of software and its dependent components on heterogeneous computing platforms directly affects its reusability. In this case, the provenance of users during the actual use of computing platforms is equally essential and effective.

Different forms of software have varying degrees of reusability, such as source code being easy to change but challenging to execute and binary files being easy to implement but hard to modify. Overall, source code is the most reusable form of software. In FyDev, we consider the re-usability of the research software of IM. The “Build” module in FyDev enhances the usability (generating executable files) of research software in IM. It supplements the building process into the context of Python runtime and supports deployment, installation from source code, and provenance of the process.

The source code is the most flexible way to manage and use research software in IM, which is determined by the characteristics and needs of research software in the fusion community. Firstly, it is easy to manage multiple versions and complex software environments. The development languages and technologies are diverse and cross-generational, such as MATLAB, Fortran, Python, C/C++, etc. This requires supporting multiple languages, versions, and even bit-numbered compilation environments in a runtime environment. Moreover, there is no unified installation and compilation method for physical code, and it depends on different basic libraries. Therefore, multiple sets of deployment environments that can be stacked and coexist are needed to run these codes. Better source code organization and management can quickly generate complex stacked environments. Secondly, it increases the flexibility of software invoked in IM. The physical components in IM are dynamically changed according to needs. Different workflows may use the same software, but other versions may be required for various problems. The entire software process can be mastered through the source code, which is convenient for switching versions.

Thus, in FyDev, metadata records the specification information of the software. The build module adds the build process of the software to the runtime environment. The build process is automatically tracked for the current runtime environment, and the results are recorded in the local description file. The build module supports the flexible building of source code and the coexistence of multiple-version software environments. Thus, the complete provenance and build module of promote software (re)use.

The FyDev implementation facilitates the migration of IM’s software environment across different sites, and the ID ensures consistency of the software’s version and software environment across sites. This relies on several key points: (1) The metadata file in FyDev acts as a source for the different sites. (2) The ID contains toolchain information that agrees with the software’s base environment. There is a one-to-one correspondence with the metadata. (3) The metadata file records the build method or builds configuration file that the build module parses to generate the required installation files. (4) FyDev is not dependent on the installation directory and can be adapted to different directory structures on different platforms.

It is important to note that the reusability described here refers primarily to how the software is used. Software developers ensure the reusability of software functions and licenses not covered by the IM system as the platform on which the software is used.

## Methods

In this section, we use GENRAY (https://compxco.com/genray.html) and CQL3D (https://compxco.com/cql3d.html) as examples to demonstrate how FyDev manages research software in IM and helps achieve the FAIR4RS principles for them. The coupled GENRAY-CQL3D code is used for systematic ray-tracing and Fokker-Planck analysis for EAST Lower Hybrid wave Current Drive (LHCD) experiments^24^. The distribution functions from CQL3D are coupled to calculations of X-ray Bremsstrahlung energy spectra along specified sight lines. Electron cyclotron microwave emission, solving the plasma wave energy transport equation along rays terminating at a detector.

**Fig. 2:**
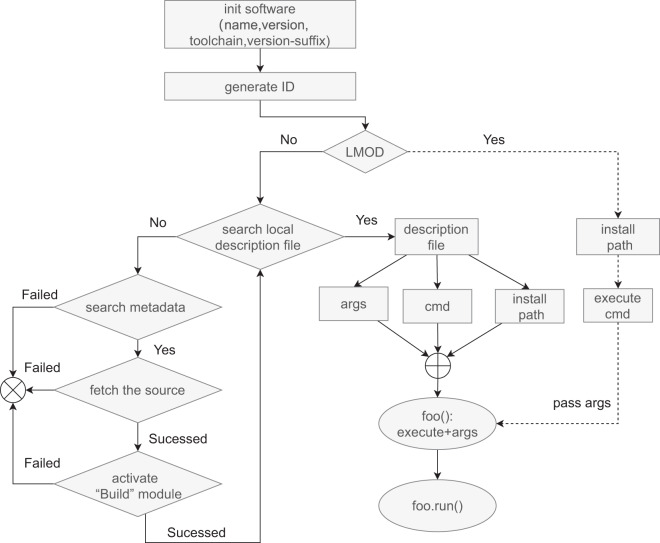
The detail execution flow in FyDev. This workflow combines IDs, metadata repositories, building blocks, and local description files into a Python callable module. In FyDev, the local description file is used as a flag to determine if the package has been deployed. If the description file exists, information such as the installation directory, run commands, interface parameters, etc., are parsed from the description file. Otherwise, The “Build” module is triggered to install it. FyDev is designed to compatibility with existing software environments in HPC.

The list in Table 4 shows the usage of research software in FyDev. The detailed execution process behind the code is given in Fig. 2. To explain the runtime, it is assumed that GENRAY is not installed and that CQL3D is an existing installation on the system but not under FyDev. The code list has the following steps.

**Table 4:** This code list shows how to use FyDev to manage software in IM.The code imports *FyRepository* to initialize FyDev. It then loads two physics software modules, GENRAY and CQL3D, and runs them with specific parameters. When software is not deployed, the “Build” module is automatically triggered to install and deploy it.

Lines 1–14 initialize the Fyrepository. They give the path to the software installation and the metadata repository. Currently, two environments are supported: (i)Traditional stack environment based on LMOD (query path based on Line 5). (ii) Stack environment based on FyDev (query description file based on ID in the path in Line 6).

For GENRAY, maintained by FyDev. Lines 16–18 show the calculation process of GENRAY. Firstly, *fydev.physics.genray* accepts user information, including *version: 201213, toolchain: gompi, version: 2020b*, and initializes the GENRAY module. An ID is generated during initialization to identify the *GENRAY: /fydev/physics/genray-201213-gompi-2020b*. The ID is used to query the software repository. The initialization phase consists of the following processes.

- First, query whether the software’s local description file exists. If it exists, return the description file. Otherwise, it proceeds to the next steps:
  - The access repository includes local or remote metadata in lines 9–13. Query the corresponding metadata file or similar template file based on the ID.
  - Obtain the source code. Find the source code based on the source information in the metadata.
  - Activate the build system. Generate the compilation and installation configuration files. The package management tool must be based on the metadata file’s build information. The path to the installation configuration file is given directly for complex builds.
  - Invoke the package management tool to compile and install the software and deploy it in the corresponding stack directory while distributing the description file in the stack directory.
- Second, it parses the description file to obtain the software environment, execution command, and parameters and combines them to generate the Python package for GENRAY.
- Third, it runs the software: *genray.run()*.

For CQL3D, maintained by traditional LMOD. Lines 20–22 show the calculation process of CQL3D. First, an ID identifying CQL3D is generated based on the information *version: 201213, toolchain: gompi, version: 2020b*, and the ID is */fydev/physics/cql3d-201213-gompi-2020b*. It uses the ID to query the stack environment in the system. It finds that CQL3D is in the LMOD stack environment. It uses LMOD to obtain the installation directory easily. Combined with the parameters passed into *bin.cql3d (dt = 0.1, input = genray_output.nc_file)*, it generates the Python package for CQL3D. Here, without CQL3D, the process fails and does not attempt to install it.

Both GENRAY and CQL3D in the examples are written in Fortran and can traditionally be directly run in the shell command line. In the IM system, Python is used as a glue language, and a unified API is needed to encapsulate and invoke the Fortran code. The example code demonstrates how the string that makes up the component ID is transformed into a callable package module in Python using FyDev. Note that the processing of GENRAY and CQL3D in the code is slightly different. GENRAY uses FyDev’s package management organization stack, while CQL3D uses the traditional LMOD package management system. This means that FyDev is a strictly independent management system compatible with the traditional package organization. The difference is that FyDev adds the building process to the description file, making the description of the software more complete, which enhances the reusability of the software.

Therefore, this article explains how FyDev, applied to IM for EAST, considers the management and invocation of software packages using Python. FyDev uses a centralized metadata repository to manage the software environment. It also uses a local “description file” to describe the research software’s state in the repository. By parsing the “description file”, FyDev obtains the software’s runtime environment, execution command, and execution parameters and combines them into a Python package. FyDev does not develop package build tools but uses distributed tools that support source code build, such as EasyBuild. FyDev documents detailed metadata information for software. FyDev follows the FAIR4RS principle in the Python context by using a unified API, standardized version management, and modular packaging to find, access, build, and invocate software.

## Data Availability

No data is being shared.

## Code availability

We use the ZENODO service to keep a persistent archive of FyDev code, with 10.5281/zenodo.8098117^33^.
